# Medical advice and diabetes self-management reported by Mexican-American, Black- and White-non-Hispanic adults across the United States

**DOI:** 10.1186/1471-2458-12-185

**Published:** 2012-03-12

**Authors:** Joan A Vaccaro, Daniel J Feaster, Sandra L Lobar, Marianna K Baum, Marcia Magnus, Fatma G Huffman

**Affiliations:** 1 Robert Stempel College of Public Health and Social Work; Department of Dietetics and Nutrition, Florida International University, Miami, FL, USA; 2 Department of Epidemiology and Public Health; Miller School of Medicine, University of Miami, 1120 NW 14th Street, Room 1055, Miami, FL 33136, USA; 3 College of Nursing and Health Sciences; Department of Nursing, Florida International University, Miami, FL, USA; 4 Department of Dietetics and Nutrition, AHC-1-435, Robert Stempel College of Public Health and Social Work, 11200 S. W. 8th Street, Miami, FL 33199, USA

**Keywords:** Medical advice, Diabetes self-management, Mexican-American, Black non-Hispanic, Race/ethnicity, Minorities

## Abstract

**Background:**

Diabetes has reached epidemic proportions in the United States, particularly among minorities, and if improperly managed can lead to medical complications and death. Healthcare providers play vital roles in communicating standards of care, which include guidance on diabetes self-management. The background of the client may play a role in the patient-provider communication process. The aim of this study was to determine the association between medical advice and diabetes self care management behaviors for a nationally representative sample of adults with diabetes. Moreover, we sought to establish whether or not race/ethnicity was a modifier for reported medical advice received and diabetes self-management behaviors.

**Methods:**

We analyzed data from 654 adults aged 21 years and over with diagnosed diabetes [130 Mexican-Americans; 224 Black non-Hispanics; and, 300 White non-Hispanics] and an additional 161 with 'undiagnosed diabetes' [N = 815(171 MA, 281 BNH and 364 WNH)] who participated in the National Health and Nutrition Examination Survey (NHANES) 2007-2008. Logistic regression models were used to evaluate whether medical advice to engage in particular self-management behaviors (reduce fat or calories, increase physical activity or exercise, and control or lose weight) predicted actually engaging in the particular behavior and whether the impact of medical advice on engaging in the behavior differed by race/ethnicity. Additional analyses examined whether these relationships were maintained when other factors potentially related to engaging in diabetes self management such as participants' diabetes education, sociodemographics and physical characteristics were controlled. Sample weights were used to account for the complex sample design.

**Results:**

Although medical advice to the patient is considered a standard of care for diabetes, approximately one-third of the sample reported not receiving dietary, weight management, or physical activity self-management advice. Participants who reported being given medical advice for each specific diabetes self-management behaviors were 4-8 times more likely to report performing the corresponding behaviors, independent of race. These results supported the ecological model with certain caveats.

**Conclusions:**

Providing standard medical advice appears to lead to diabetes self-management behaviors as reported by adults across the United States. Moreover, it does not appear that race/ethnicity influenced reporting performance of the standard diabetes self-management behavior. Longitudinal studies evaluating patient-provider communication, medical advice and diabetes self-management behaviors are needed to clarify our findings.

## Background

Diabetes leads to complications such as heart disease and stroke, high blood pressure, blindness, kidney disease and nervous system disease; the risk of death for persons with diabetes is twice that of persons without diabetes [1]. Type 2 diabetes, the most common form (90-95% of all cases), has increased in the general population [2] and disproportionately among minorities (particularly African Americans and Hispanics) [3]. Mexican Americans have the highest rate of diabetes among Hispanics and are 1.7 times more likely to have diabetes as White non-Hispanics [1] and their age-adjusted diabetes death rate was 1.5 greater than White non-Hispanics [4]. African Americans are 2.1 times more likely to be diagnosed with diabetes than White non-Hispanics [1] and their age-adjusted death rate from diabetes was nearly twice that of White non-Hispanics (45.1 versus 23.3 per 100,000) [4].

Due to the many health consequences of diabetes and the nature of the disease, diabetes care is vital to quality of life and survival. Diabetes is a disease that can be managed by the individual with appropriate guidance. Yet fewer than 60% of all adults age 40 and over with diagnosed diabetes have their blood glucose, cholesterol, or blood pressure within the recommended levels for adequate control [5]. According to the American Diabetes Association's (ADA) *Standards of Medical Care *[6] an operational definition of high quality health care for persons with diabetes would include guidance on risk factor control for all of the following: 1) dietary intake and weight management; 2) glycemic and lipid control; and 3) foot and eye care.

**Figure 1:**
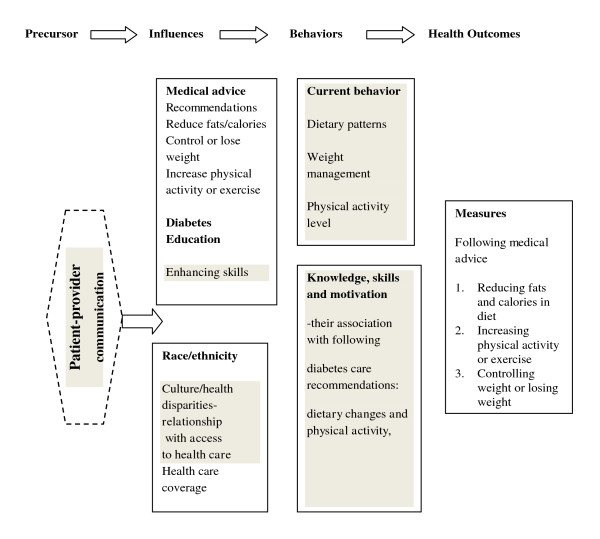
**Ecological model applied to diabetes self-management and health outcomes**. Adapted from the NHLBI workshop on predictors of obesity, weight gain, diet and physical activity; August 4-5, 2004; Bethesda MD and from Ecological model [7]. Notes: The grey areas denote constructs that are not measured by this study. The level and quality of patient-provider communication is unknown and is designated as a precursor for this study. It is assumed that persons diagnosed with diabetes would have some level of communication with health care professionals.

The ecological model (Figure 1) was used in this study as a framework for the concepts applied to DSM outcomes [7]. Medical advice, diabetes education, and the patient's race and cultural environment influence their behaviors and consequently their health outcomes. Diabetes care is largely the responsibility of the individual; however, healthcare professionals can play vital roles in the patient's skill development through effective patient-provider communication with respect to collaborative goal setting and individual assessment [8,9].

## Methods

### Data collection

All data used for this study were openly, publically available [10]. Raw data were extracted from datasets from the National Health and Nutrition Examination Survey, 2007-2008 (NHANES 2007-2008) [10]. This survey contained data for 10,149 individuals of all ages. Data were collected between January 2007 and December 2008 using a complex, multistage, probability sample design. The data from NHANES consist of 2-year representative samples of the non-institutionalized, civilian U.S. population. All participants read, understand and sign informed consent forms based on the approved study protocol from the National Center for Health Statistics Research Ethics Review Board [10]. The study was limited to male and female adults ages ≥ 21 years who provided self-report diagnosis of diabetes or who had hemoglobin A_1c _(A1C) ≥ 6.5, underwent an examination at the Mobile Examination Center (MEC), and belonged to one of the following race/ethnicity classifications: Black non-Hispanics (BNH), Mexican American (MA), and White non-Hispanics (WNH). Of the total sample size for the 2007-2008 participants, there were 2,064 MA, 1,147 other Hispanics, 2,141 BNH, 3969 WNH and 441 persons classified as "other". From the combined sample, there were 777 persons (7.7%) of the 9372 valid cases who responded to the screening question for diabetes diagnosis. The categories for "other Hispanics" and "other races" were 10.8% and 2.7%, respectively and did not have a sufficient sample sizes for comparative analyses. Approximately 2% (n = 14) of the subjects were minors (< 18 years). The final sample size of participants who met the inclusion criteria for the main study question was [N = 654 (130 MA, 224 BNH and 300 WNH)]. There were n = 161 classified as 'undiagnosed diabetes'. The final sample size, including individuals with 'undiagnosed diabetes', was [N = 815(171 MA, 281 BNH and 364 WNH)].

### Definitions

#### Diagnosed diabetes

Several questions were considered for the construction of the variable 'diagnosed diabetes': 1) "The next questions are about specific medical conditions. Other than during preganncy, have you ever been told by a doctor or health professional that you had diabetes or sugar diabetes?"; 2) "How old were you when a doctor or other health professional first told you that you had diabetes or sugar diabetes?"; and 3) "When was your diabetes diagnosed?"

Each question was by self-report and subject to recall-bias. Since there were missing values for questions 2 and 3, construction of the variable, 'diagnosed diabetes' was based on question 1.

#### Undiagnosed diabetes

Both fasting blood glucose (FBG) and hemoglobin A_1C _(A1C) were initially considered for construction of the 'undiagnosed diabetes' variable. The latter was chosen, since there were approximately twice as many missing values for FBG as compared to A1C. The inclusion criteria were the same as for 'diagnosed diabetes' with the exception that both conditions were met: 1) 'no self-report of being diagnosed with diabetes'; and, 2) percent A1C ≥ 6.5.

### Ecological model

The ecological model suggests that changes in knowledge, skills and attitudes will change behaviors and consequently health outcomes. Behaviors that are hypothesized to impact DSM health outcomes were measured in the NHANES [10] and in the applied DSM model using the 'current behaviors' (dietary plans, weight management, and physical activity changes). Patient provider communication is an assumption of the model and it was not measured in this study.

### Measurements using the ecological model

#### Independent variables

Medical advice (on diet, weight management and physical activity and 'told by a doctor or health provider in the past 12 months'), fits into the ecological model as an environmental influence which acts on the individual and helps to promote health behavior change. Influences were measured in NHANES [10] and in the applied model (Figure 1) using 1) 'medical advice' (reduction of fat or calories, control or loss of weight, increased physical activity or exercise); 2) 'diabetes education' (enhancing skills related to self-care); and, 3) 'race/ethnicity' (culture/health disparities). The NHANES database [10] measures reported 'medical advice' without making a distinction among the healthcare provider (the response choice for these questions were 'told by a doctor or health provider'). The response for diabetes education was 'When was the last time you saw a diabetes nurse educator or dietitian or nutritionist. Do not include doctors or other health professionals'.

#### Dependent variables

'Health Outcomes' measured in this study were the changes in behavior related to diabetes self-management (reporting reducing fat or calories in the diet, increasing physical activity or exercise and controlling or losing weight) (Figure 1). Questions on medical advice were used for the first time by NHANES [10] and correspond to recommendations from the *Global Guideline for Type 2 Diabetes*, lifestyle management standard care [11]. Medical advice questions examined in this study all were phrased with the standard language 'to lower your risk for certain diseases, during the past 12 months have you ever been told by a doctor or health professional to' and the advice added for each question were 1) reduce the amount of fat or calories in your diet; 2) increase physical activity or exercise; and, 3) control weight or lose weight. The health behavior questions were phrased 'To lower your risk for certain diseases, are you now doing any of the following' and behavior added for each question were: 1) reducing the amount of fat or calories in your diet; 2) increasing your physical activity or exercise; and, 3) controlling weight or losing weight. A variable for recent diabetes education was created with two categories (two years or less and more than 2 years or never) based on responses from the following 'When was the last time you saw a diabetes nurse educator or dietitian or nutritionist for your diabetes? Do not include doctors or other health professionals'.

Logistic regression models were performed with reporting receipt of medical advice within the past 12 months by race/ethnicity for each outcome of diabetes self-management behavior. Specifically, the independent variable for model 1 was binary responses to 'told to reduce fat or calories' and the dependent variable was 'currently reducing fat or calories'. Similarly, model 2 assessed the likelihood of 'told to increase physical activity' with 'currently increasing physical activity' and model 3 contained 'told to control or reduce weight' as the independent variable and 'currently controlling or reducing weight' as the dependent variable. Full models contained age, gender, education, health insurance, overweight/obese, and diabetes education.

#### Additional analysis dependent variable: obesity

A binary variable was constructed using a proxy measure of obesity, body mass index (BMI). Classification was ≥ 30 for 'obese' and < 30 'not obese' with measurements of weight in kg divided by height in m^2 ^(kg/m^2^). Values used for BMI for this study were calculated by direct measurements taken in the MEC by NHANES. Next, quartiles of BMI were compared with each medical advice and behavior by the Chi Square Test.

#### Study questions

In this study, we assessed the following.

1. Whether the effect of medical advice on the likelihood of performing the corresponding recommendations differs by race/ethnicity.

2. Whether individuals having 'undiagnosed diabetes' (no self-report of being diagnosed with diabetes and a percent hemoglobin A1C ≥ 6.5) versus 'diagnosed diabetes' (self-report of being diagnosed by a doctor or health professional) would differ in the relationship between given medical advice (yes/no) and its effect on the corresponding behaviors.

3. Will the effect of reporting being told to control or loss weight by reporting performing the behavior (controlling or losing weight) be associated with obesity.

### Data analysis

Sample weights were constructed and included in the data sets to account for complex sample design and achieve unbiased national estimates. The choice of sample weight was based on the data file with the smallest sample size as recommended by the National Center for Health Statistics (NCHS) guidelines [12]. Data analysis was conducted with IBM-SPSS version 18 with a complex sampling add-on. Prior to analysis, continuous variables were assessed for normality by Q-Q plots and when needed, transformed. Post-analysis, continuous variables were tested by residual graphs for skew. Hierarchical logistic regression models were conducted for medical advice by race/ethnicity predicting adequate/inadequate DSM adding variables associated by the literature as covariates. We examined 3 different types of medical advice, therefore a Bonferroni correction was used to ensure an overall error rate of 0.05 and p < 0.017 was considered significant. Models were estimated with and without covariates. Covariates considered included age, gender, health insurance, diabetes education and education. In addition, obesity was added for 'told to reduce fat or calories' and 'told to increase physical activity; whereas overweight and obesity was included for 'told to control or loss weight'. The final models retain covariates with p-values of ≤ 0.2 for diagnosed diabetes. Undiagnosed diabetes models were adjusted for health insurance, age, gender, and body mass index categories. The Wald F statistic was used to determine model significance for complex logistic regression analysis [13] where the degrees of freedom are constrained to a constant value (the primary sampling units minus the strata which equal 17 for this data). Results are only presented for models which significantly predicted the outcomes (values available upon request). Odds Ratio (OR) and 95% confidence limits are presented for the model without covariates and adjusted odds ratio (AOR) and 95% confidence limits are presented for the models with covariates. Where there was information missing, list-wise deletion was used and the number for each analysis was provided.

## Results

### Population characteristics

The general characteristics of the participants are shown in Table 1. Over two-thirds of the sample was classified as obese and 88% were classified as overweight based on their body mass index (BMI). There were significant differences among race/ethnicity for age and education. White non-Hispanics were approximately 4 years older than BNH and MA. There was not a significant difference in reporting having health coverage in the past 12 months between BNH (85.2%) and WNH (93.1%) (p = 0.055). However, MA were significantly less likely to report having health coverage (62%). In addition, over 40% of MA responded they did not know how many times per year they saw their doctor; whereas approximately half of BNH and WNH reported they did not recall the number of doctor's visits over the past year. Of those who reported number of doctor visits, there were no significant differences among participants by race/ethnicity; however, those reporting specific frequencies may not be representative of their group (data not shown). These results indicated there were significant differences in access to health care in MA as compared to WNH. Approximately one-third of the combined sample reported not receiving advice to reduce fat/calories, increase physical activity/exercise or control/lose weight. Additionally, participants who were overweight or obese were four times more likely to report receiving advice to control or reduce their weight than individuals with a normal body mass index (data not shown).

**Table 1:** Characteristics of the participants (N = 624)a

Variable^b^	MA	BNH	WNH	*P *_MA/WNH_	*P *_BNH/WNH_	*P *_Total_
Age (years)	56.2 ± 1.95	57.6 ± 0.89	60.7 ± 0.65	0.019	0.012	0.002

Gender				-	-	0.127

Male	54 (48.1)	96 (398)	160 (50.2)	-	-	

Female	67 (51.9)	117 (60.2)	128 (49.8)	-	-	

Years with diabetes	9.68 ± 0.85	11.6 ± 0.67	11.6 ± 0.68	0.127	0.989	0.242

Education						< 0.001

≤ 8^th ^grade	56 (41.5)	25 (9.2)	37 (10.1)	-	-	-

> 8^th ^< HS	29 (25.7)	60 (28.2)	59 (15.0)	-	-	-

HS/GED	12 (11.8)	50 (24.1)	89 (31.0)	-	-	-

Some college	23 (21.0)	78 (38.6)	103 (43.7)	-	-	-

Income						0.132

< 15,000	21 (17.0)	47 (21.3)	49 (12.0)	-	-	-

15 to 34,999	29 (26.5)	62 (30.9)	111(34.2)	-	-	-

35 to 54,999	26(24.0)	33(15.8)	41(16.4)	-	-	-

55 to 74,999	9 (7.1)	24 (12.3)	21 (12.7)	-	-	-

≥ 75,000	20 (15.3)	28 (16.1)	38 (22.4)	-	-	-

Refused	5 (3.4)	2 (0.6)	4 (2.2)	-	-	-

Don't know	6 (3.9)	5 (2.9)	2 (0.6)	-		

Health insurance^c ^*none in the past 12 months*	38.0 (6.7)	14.8 (3.3)	6.9 (1.2)	< 0.001	0.055	< 0.001

Age first told had diabetes^d^	71.8(21)	46.0(1.1)	60.8(5.4)			< 0.001

*Abbreviations: *MA = Mexican American; BNH = Black non-Hispanic; WNH = White non-Hispanic; BMI = body mass index; LDL = low-density lipoprotein cholesterol.

^a^unweighted cases varies based on the variable for the: MEC (mobile examination center) participants.

^b ^Continuous variables are given as (mean ± SE) were tested by one-way ANOVA and categorical; variables are given as N (%) and were tested by Pearson's chi-square.

^c ^The values are percent (*SE*) for the *unadjusted *odds ratios. The *adjusted *odds ratios are as follows; OR _MA/WNH _= 5.73 (2.17, 15.1), *p *< 0.001; OR _BNH/WNH _= 1.90 (0.77, 4.70), *p *= 0.151 (controlling for age and education).

^d ^Results are mean(SE). N = 622 (32 (5%) missing responses).

### Medical advice, race/ethnicity, and behavior

The Odds Ratios for receiving medical advice and following medical advice are presented in Table 2 for participants with diagnosed and undiagnosed diabetes. There was a significantly greater chance of performing the recommended behaviors if given the advice, independent of ethnicity/race. Ethnicity/race did not differ with respect to receiving advice and following it, neither as a main effect nor as an interaction.

**Table 2:** Likelihood of receiving medical advice by race/ethnicity and performing the behaviors

Dependent (changed behaviors)	Independent Medical advice	Unadjusted OR (95% CI)	Adjusted OR (95% CI)
Diagnosed diabetes			
Reducing fat or calories^a^	Told to reduce fat or calories	8.78(5.57, 13.8)	6.87(3.83, 12.3)
Increasing physical activity or exercise^b^	Told to increase physical activity or exercise	6.53 (3.73, 11.4)	6.34(3.55, 11.5)
Controlling or losing weight^c^	Told to control or lose weight	4.64 (2.32, 9.32)	4.13(1.98,8.62)

Comparison undiagnosed/diagnosed diabetes^d^			
Reducing fat or calories	Told to reduce fat or calories		

	Diagnosed	8.73(5.86, 13.0)	8.58 (5.78, 12.7)
	Undiagnosed	2.54 (0.68, 9.43)	2.28 (0.55, 9.53)

Increasing physical activity or exercise	Told to increase physical activity or exercise		

	Diagnosed	6.29 (3.88, 10.2)	6.72 (3.87, 11.7)
	Undiagnosed	3.03 (1.47, 6.27)	2.87 (1.34, 6.12)

Controlling or losing weight	Told to control or lose weight		

	Diagnosed	4.48 (2.70, 7.44)	4.18 (1.96, 8.94)
	Undiagnosed	2.88 (0.52, 16.07)	2.42 (0.38,15.16)

^a^Adjusted for age, gender, obesity and diabetes education; ^b^Adjusted for age; ^c^Adjusted for overweight/obesity; ^d ^Unadjusted models include the main effect of ethnicity/race; covariates for the adjusted models include age, gender, race/ethnicity, body mass index category, and health insurance.

*Notes*: The Odds are for those performed the behavior and the ratio is 'received/did not receive' the advice.There were no statistically significant differences comparing undiagnosed/diagnosed by told for any model.

#### Diagnosed diabetes

##### Reporting 'told to reduce fat or calories'

Being told by a medical provider to reduce fat or caloric intake was significantly associated with an increased likelihood of reporting having reduced fat or calories in both unadjusted OR = 8.78 (5.57, 13.8)and in adjusted models AOR = 6.87 (3.83, 12.3). The final covariates in the model were age (p = 0.142), gender (p = 0.062), obesity (p = 0.166), and diabetes education (p = 0.110). The relationship was not modified by race/ethnicity. Race/ethnicity was not significant with (p = 0.148) or without (p = 0.284) covariates. Neither was the interaction, 'told by race/ethnicity' significant for any model (p = 0.344), no covariates; (p = 0.428), with covariates.

##### Reporting 'told to increase physical activity or exercise

Respondents who reported that they had been told by a medical provider (physician or other healthcare provider) to increase physical activity or exercise were significantly more likely to report having increased their physical activity or exercise in both the unadjusted OR = 6.53 (3.73, 11.4) and adjusted models AOR = 6.34 (3.55, 11.5). The only covariates that remained in the adjusted model was age (p = 0.011). There was no effect of race/ethnicity on this relationship. Neither race/ethnicity [unadjusted (p = 0.866); adjusted (p = 0.906)] nor the interaction of 'told by race/ethnicity', were statistically significant [unadjusted (p = 0.916); adjusted (p = 0.933)].

##### Reporting 'told to control or lose weight'

Respondents who reported having been told to control or lose weight were approximately four times more likely to report having controlled or lost weight than those who did not report being told, whether examining the unadjusted [OR = 4.64 (2.32, 9.32)] or adjusted Odds Ratio [AOR = 4.13 (1.98, 8.62)]. Being overweight was the only covariate that remained in the adjusted model. There was no impact of race/ethnicity on this relationship. Neither the main effect for race/ethnicity [unadjusted (p = 0.867); adjusted (p = 0.849)], nor the interaction of 'told by race/ethnicity' were statistically significant [unadjusted (p = 0.610); adjusted (p = 0.688)].

#### Undiagnosed diabetes versus diagnosed diabetes

Medical advice variables in this study were not specific to those with a diabetes diagnosis.. Exploratory chi-square analyses revealed approximately half the participants with 'undiagnosed diabetes' did receive medical advice for weight [46.9 (33.4, 61.0), SE = 6.6]; physical activity [52.7 (41.3, 63.9), SE = 5.4]; and, fat/calories [44.3 (31.9, 57.4), SE = 6.1]. The percent of 'undiagnosed' receiving the advice was lower than for diagnosed cases where approximately two-thirds reported receiving medical advice for weight [63.3 (57.7, 66.6), SE = 2.1]; physical activity [66.5 (60.0, 72.5), SE = 3.0]; and fat/calories [65.6(61.4, 69.7), SE = 2.0]. Receiving medical advice for individuals who were 'undiagnosed was higher than for the general population (20-27%) (data not shown). Table 2 presents the OR and AOR for performing the recommended behavior if given the advice for diagnosed (top of table) and undiagnosed compared with diagnosed (bottom of table). Diagnosis status was not related to engaging in any of the health behaviors for the unadjusted [p_lose wt _= 0.762; p_reduce fat _= 0.560; p_increase PA _= 0.740] or adjusted [p_lose wt _= 0.600; p_reduce fat _= 0.253; p_increase PA _= 0.690] models. The interactions of diagnosis status and being told to engage in the health behavior were also not related to engaging in the behavior for the unadjusted [p_lose wt _= 0.655; p_reduce fat _= 0.109; p_increase PA _= 0.102] or the adjusted [p_lose wt _= 0.596; p_reduce fat _= 0.100; p_increase PA _= 0.096] models. Although these interactions are not statistically significant the OR/AOR are uniformly smaller for the undiagnosed in each medical advice model.

#### Additional analysis: interaction of medical advice for weight management by following the advice with obesity

The unadjusted logistic regression model with obesity as the dependent variable included reported medical advice for weight loss, reported weight loss, the interaction of medical advice by weight loss and race as the effects. The main effects for '*told to control or lose weight' *was significant in the unadjusted (p < 0.001) and the adjusted models (p < 0.001); where the main effect for having lost weight was not statistically significant [unadjusted (p = 0.432) and adjusted (p = 0.452)]. The interaction of being told and losing weight was associated with obesity [(p = 0.009) unadjusted; (p = 0.023) adjusted]. Race was not significant in the models [(p = 0.474) unadjusted; (p = 0.263) adjusted]. The resulting odds ratios for the four groups showed that for the group of participants who had not lost weight, the odds of being obese if told to lose weight relative to not being told to lose weight were the highest of the subgroups [OR = 17.7(7.06, 44.2); AOR = 16.5 (5.95, 45.5)]. For those who actually had lost weight, odds of obesity for those who had been told were 4.31 (2.83, 6.02), unadjusted and 4.74(3.09, 7.29), adjusted, relative to those who had not been told. For the subgroup who had not been told to lose weight, those who had actually lost weigh had higher odds of being obese than those who had not lost weight [OR = 2.70 (1.33, 5.980); AOR = 2.43 (1.10, 5.36)]. Only for the subgroup who had been told to lose weight was the act of actually losing weight associated with lower likelihood of obesity, though these odds were not significantly different from 1 [OR = 0.66 (0.26,1.68); AOR = 0.70 (0.28,1.77)]. Age, gender, education and health insurance were tested for the adjusted model. Education and health insurance were removed to achieve model fit, leaving age and gender as the covariates in the adjusted model.

#### BMI, medical advice and behavior

Individuals who received medical advice were more likely to be in the upper quartiles of the BMI distribution for each medical advice. Similarly individuals who reported having performed the behavior were also more likely to have been in the upper quartiles of the BMI distribution for reducing fat or calories and losing weight, but not for increasing physical activity. The median BMI within the groups who had and had not received medical advice and engaged in the behavior is shown in Table 3 to illustrate these results.

**Table 3:** Median, 25^th ^and 75^th ^percentile BMI stratified by level of medical advice and behavior

Medical advice	Median BMI (P25, P75)		*P*-value
	**YES**	**NO**	

Told to reduce fat or calories	33.5 (29.7, 38.8)	29.1 (26.0, 33.1)	< 0.001
Told to increase physical activity or exercise	33.5 (29.4, 38.5)	29.1 (25.9, 32.8)	< 0.001
Told to control or lose weight	34.3 (30.5, 39.3)	28.2 (25.7, 31.8)	< 0.001

**Behavior**	**Median BMI (P25, P75)**		***P*-value**

	**YES**	**NO**	

Reducing fat or calories	32.3 (28.7, 37.6)	29.6 (26.1, 34.7)	0.002
Increasing physical activity or exercise	32.4 (28.1, 37.2)	30.7 (26.9, 36.1)	0.200
Controlling or losing weight	32.3 (28.8, 37.3)	29.7 (26.0, 34.9)	< 0.001

Cross-tabs for each medical advice and each behavior with quartiles of BMI were performed by complex analysis. Significance was based on the adjusted F and its degrees of freedom (adjusted for complex design). The adjusted F is a variant of the second-order Rao-Scott adjusted Chi-Square statistic.

## Discussion

The ecological model applied to this study was supported with certain limitations. Our result indicated that, regardless of race/ethnicity, individuals with diabetes who reported being given medical advice to perform essential DSM behaviors: reducing fat or calories; increasing physical activity or exercise; and, controlling or losing weight, were more likely to report performing the corresponding behavior than those who were not told. Weight reduction and management, an important aspect of DSM, can be achieved by reducing fat or calories and increasing physical activity. Performing these skills is central to the recommendations for persons with type 2 diabetes by the ADA [6].

Despite the recommendation by ADA that all persons with diabetes be given this medical advice, approximately one-third of the combined sample reported not receiving advice to reduce fat/calories, increase physical activity/exercise or control/lose weight. Participants in the normal weight category were four times less likely to report receiving advice to control or reduce their weight as compared to individuals in the overweight or obese category. Understanding the context of medical advice and diabetes care within the ecological model may be of value for understanding these discrepancies.

Diabetes is a public health problem requiring a multilevel systems approach for prevention and treatment [14]. The population-based approach advocated by Glasgow et al [14] includes personal, family, health care team, and community influences that impact the promotion or inhibition of diabetes self-management and lifestyle changes. A key factor, interwoven through each system, is communication. Investigations concerning the relationship between patient-provider communication and health behavior were conducted in the late 1960's [15]. There have been detailed protocols for medical advice, which included collaborative goal setting, in the field of nursing since the 1960's. Despite these advances, process and outcome evaluation of patient-provider communication remains underdeveloped. The complex dynamics of interpersonal relationships makes assessment of 'culturally sensitive and collaborative,' patient- provider communication difficult. Moreover, few studies have investigated whether the message was received in the manner it was intended for diabetes patients and if race/ethnicity affected the communication process.

Several studies have indicated health disparities by race/ethnicity have occurred in participatory provider-patient relationships [16-19]. There is some evidence that improvements in diabetes outcomes may not occur for minority patients, even when physicians are made aware of racial disparity in diabetes care and outcomes [20]. A 12-month, randomized controlled trial applying cultural competency training found no improvements in diabetes outcomes, despite the physicians' increased awareness of health disparities [20]. For this study, it was conceivable that some patients received the standard, recommended advice but did not process the information. We are not sure to what degree the communication was received as intended and if there were variations by race/ethnicity. Consequently, patient-provider communication may have been a confounder in determining whether receiving medical advice resulted in the corresponding DSM behaviors. Since patient-provider communication was not measured but may have influenced health outcomes, it was considered a precursor for this study's theoretical model.

With respect to our theoretical model's categories of current behavior leading to health outcomes, several points need to be clarified. First, medical advice given was measured by self reporting. The communication process, together with the patient's health beliefs and personal characteristics (knowledge, motivation, and self-efficacy) were factors in determining if the advice given was understood as it was intended. Second, medical advice may not be the driving force changing behaviors to the corresponding desired health outcomes. It is not known if the participants were performing these dietary and physical activity behaviors prior to being given medical advice. The inclusion of questions regarding the connection of advice with the corresponding behaviors by subsequent NHANES would be needed to clarify their associations.

Another difficulty in assessing the effectiveness of medical advice was the nature of the questions. Medical advice and health behavior questions asked by NHANES were broad. The recommendations for lifestyle management comprehensive care includes this advice; albeit with individual advice on diet and physical activity as well as ongoing counseling and access to a dietitian or healthcare professional trained in nutrition. The questions did not specify the clinical profession who gave the advice (such as physician, nurse, nurse practitioner, or dietitian) or the quality and frequency of the advice.

### Strengths and limitations

There were several limitations of this study. First, cause and effect could not be established by this study since the data were comparing groups from a single time point. Second, there may have been subject bias in some of the variables. The demographic data and data concerning medical advice received were self-reported. It may be that those participants who followed advice were more likely to remember being given the advice. Third, the comparisons by race/ethnicity were not of completely homogenous groups. Within the category "Black, non-Hispanic" several Caribbean cultures were combined with African American. Immigrant minorities (Haitian versus English-speaking, Caribbean Blacks) are likely to have acculturation and health belief differences from non-immigrant minorities (African Americans). Within the "Mexican American" classification differences in length of time in the United States accounted for variation of homogeneity. Even though NHANES over-samples the poor for each racial group, and the variable education level was chosen as a control, income could not be completely equalized across groups. Fourth, there were variations in exposure variables. While the major exposure variables for medical advice were standard question, their interpretation may vary by the individual or across race/ethnicities. For example, the quantity and quality of the specific dietary and physical activity recommendations were not asked and interpretation may vary by race/ethnicity. Comparably, diabetes education varied by frequency (within the past two years) and duration (contact time with the diabetes educator) and may have differed in quality. It was possible that the exposure to diabetes education could have been unequal across race/ethnicity. Finally, the comparisons of the diagnosed and undiagnosed (based on A1C) were of relatively low power and whereas no statistically significant impact was found, there were clinically important differences in the estimated Odds Ratios for these two groups. Further examination of individuals with undiagnosed probable diabetes is warranted.

One limitation of this study was the limited data inherent in all secondary analysis research. In particular, data regarding the patient-provider communication processes were absent in the NHANES database; hence in this study. It has been well-documented that the patients' participation in treatment goals improves health outcomes. Despite the limitations, a major strength of this study was the use of a national database (NHANES), which has specialized in collecting health data by race/ethnicity. Since this was the first year that NHANES [10] included data concerning medical advice for DSM; this study was one of the first to use a national database to assess health disparities of reported medical recommendations.

## Conclusions

This study has demonstrated the association of specific medical advice and the self-management behaviors for three nationally represented racial/ethnic groups with diabetes. Although the literature indicates race/ethnic differences in health behavior, these findings suggest diabetes self-management may be influenced by medical advice, independent of race/ethnicity. It is recommended that healthcare providers reinforce specific weight management advice for patients at every opportunity. Case studies and longitudinal investigations are needed to determine causality among quality of patient-provider communication, standards of care received, and corresponding behaviors.

## Abbreviations

ADA: American Diabetes Association; AHRQ: Agency for Healthcare Research and Quality; AOR: Adjusted odds ratio; BNH: Black non-Hispanic; BMI: Body mass index; CDC: Center for Disease Control and Prevention; DSM: Diabetes self-management; MA: Mexican-American; MEC: Mobile Examination Center; NDSS: National Diabetes Surveillance System; NIDDK: National Institute of Diabetes and Digestive Kidney Diseases; NCHS: National Center for Health Statistics; NHANES: National Health and Nutrition Examination Survey; OR: Odds Ratio; WHN: White non-Hispanic.

## Competing interests

The authors declare that they have no competing interests.

## Authors' contributions

JAV participated in designing the study, acquiring the data, analysis and writing of the first draft. SLL contributed to the conceptual model. MKB and FGH made substantial contributions to the methodology. DJF participated in critical review of the statistics. MM integrated the public health perspective into the study. All authors made substantial contributions to the conception and design, interpretation of data, and critically revised the intellectual content of the manuscript. All authors have approved the final version of the manuscript.

## Pre-publication history

The pre-publication history for this paper can be accessed here:

http://www.biomedcentral.com/1471-2458/12/185/prepub
